# Conjugated Polymers-Based Biosensors for Virus Detection: Lessons from COVID-19

**DOI:** 10.3390/bios12090748

**Published:** 2022-09-10

**Authors:** Vinh Van Tran

**Affiliations:** Laser and Thermal Engineering Laboratory, Department of Mechanical Engineering, Gachon University, Seongnam 13120, Korea; vanvinhkhmtk30@gmail.com

**Keywords:** conjugated polymers, organic field-effect transistors, electrochemical biosensors, COVID-19 detection, DNA/RNA diagnostics

## Abstract

Human beings continue to endure the coronavirus disease (COVID-19) pandemic, which has spread throughout the world and significantly affected all countries and territories, causing a socioeconomic crunch. Human pathogenic viruses are considered a global burden for public health, both in the present and the future. Therefore, the early and accurate diagnosis of viruses has been and still is critical and should be accorded a degree of priority that is equivalent to vaccinations and drugs. We have opened a Special Issue titled “Conjugated polymers-based biosensors for virus detection”. This editorial seeks to emphasize the importance and potential of conjugated polymers in the design and development of biosensors. Furthermore, we briefly provide an overview, scientific evidence, and opinions on promising strategies for the development of CP-based electrochemical biosensors for virus detection.

Viruses are small, nanometer-scale carriers of genetic material, which can be ubiquitous in the environment, foods, animals, plants, or even in the human body; they can induce pathogens, but the majority of viruses are not enemies or killers [1]. Viruses are mainly classified into two types: DNA- and RNA- viruses, based on the nucleic acids in their genomes [2]. DNA viruses, including single-stranded DNA (ssDNA) or double-stranded DNA (dsDNA), are typically more benign than RNA viruses, while RNA viruses have a high genetic flexibility and a high rate of mutations because they present sloppiness in the copying process of their genetic code. Thus, RNA viruses can rapidly evolve and induce more pathogens compared to DNA viruses. In recent decades, various RNA viruses have appeared and caused diseases in human beings, including severe acute respiratory syndrome (SARS); influenza; hepatitis A, C, D, and E; dengue; Ebola; Middle East respiratory syndrome (MERS); Zika; human immunodeficiency virus (HIV); and other viruses. To date, more than 100 families of viruses have been identified, including both DNA- and RNA- viruses. However, current technologies have mainly concentrated on the diagnostics of RNA viruses, which exhibit a high tendency for pandemic spread.

In general, a high load of viruses can be found in the stool, saliva, respiratory droplets, and aerosols of infected patients, and can then introduce contamination to the environment; only a low viral dose is required to infect and cause disease in humans. Moreover, viruses can survive and resist environmental stressors, i.e., temperature, humidity, pH, organic loads, sunlight, and even disinfectants, which results in high transmission and infection [3]. Therefore, human pathogenic viruses are considered a global burden for both public health and the economy. Take the novel coronavirus 2019 (COVID-19) for example—almost 600 million cases have been confirmed throughout the world, with more than 6.4 million deaths reported as of August 2022 since the time of the outbreak in December 2019 [4]. Several vaccines have been developed and recently administered around the world. Although the vaccines have proven efficacious in preventing the spread of the COVID-19 pandemic, their long-term protection has not been confirmed to date. Therefore, nonpharmaceutical interventions such as social distancing and mask wearing have been widely employed as effective safeguards [5]. Since there have been no specific drugs or vaccines that can effectively and completely inhibit COVID-19, early diagnosis and management remain crucial approaches for the campaign against the spread of the pandemic. Highly specific and sensitive virus detection plays a vital role in the accurate and early diagnosis of infected patients and their isolation from the community. Many studies have been conducted to identify and detect the severe acute respiratory syndrome-coronavirus-2 (SARS-CoV-2), the virus that causes COVID-19; however, diagnosis technologies have been limited by their high cost or time-consuming nature [6]. The current standard technologies, i.e., reverse-transcription polymerase chain reaction (RT-PCR), enzyme-linked immunosorbent assays (ELISAs), computed tomography (CT), and serological assays show practical limitations in the case of massive and rapid testing. Therefore, the use of low-cost biosensors has recently been regarded as a fast and reliable alternative for virus detection [7]. In addition, biosensors allow the development of advanced technologies with rapid, accurate, portable, and large-scale features for the detection of SARS-CoV-2, as well as other viruses.

Biosensors are defined as analytical devices comprising three major modules: a sensing bioreceptor, a transducer, and a detector with a digital output [8]. In principle, the bioreceptor interacts with virus biomarkers such as RNA, proteins, or whole virus, and these interactions are then recognized and recorded by the detector module. Based on the working principle of the transducer, biosensors can be classified into three main types that can be used for virus detection: electrochemical biosensors, piezoelectric biosensors, and optical biosensors [9]. Among them, electrochemical biosensors show several advantages, such as rapid response and real-time monitoring, easy and friendly operation, high sensing performance (sensitivity and specificity), portability, and compact size [10]. These days, many studies have been conducted with the aim of further improving the sensing performance and stability of electrochemical biosensors. The discovery and development of novel materials for the increase in the interaction and recognition between biosensor probes and virus biomarkers has been one of the most promising approaches and has attracted great interest [11,12]. In the past decades, a number of advanced materials have been successfully fabricated and developed for the design of electrochemical biosensors, such as gold nanoparticles, carbon, graphene, graphene oxides, metal oxides, and carbon nanotubes [13,14]. Recently, conjugated polymers (CPs) have been proven as potential materials for electro-chemical biosensors, with excellent sensitivity and selectivity for specific biological molecules and fast electrical signals due to their unique π orbital structure and chain conformation alterations [15,16]. Furthermore, CP-based electrochemical biosensors are able to serve as multisensors, mobile biosensors, and wearable biosensors, which can facilitate the development of point-of-care (POC) systems and home-use biosensors for the detection of COVID-19 and other viruses [17]. Nonetheless, the practical applications of CP-based electro-chemical biosensors for virus diagnosis have suffered huge challenges due to the degradation, low crystallinity, and charge transport properties of pristine CPs. In order to resolve these issues, several promising strategies have been proposed for the development of CP-based electrochemical biosensors for the detection of COVID-19, as well as other viruses.

In this editorial, we would like to briefly introduce some important development strategies of CPs in biosensors for virus detection. By taking advantage of these strategies, CPs can be widely used to design flexible and wearable biosensors at local hospitals, doctors’ offices, and even households for the detection of COVID-19, as well as other viruses in the near future, which significantly contributes to community protection. The potential strategies are summarized in Figure 1. For improving the properties of CP-based biosensors, the modification and functionalization of pristine CPs are considered a key strategy for the development of electrochemical biosensors in virus detection. Functionalized CPs that are integrated in biosensors can produce a covalent attachment to improve the activity of recognition probes [18]. Due to the presence of a number of intrinsic redox states, CPs also show excellent processability after functionalization, which can improve the applicability of biosensors in practical applications [19]. CPs can be functionalized via two main approaches: (i) the functional entity can be coupled into the monomer during the synthesis process; (ii) the targeted functionality is incorporated in the monomer unit by post-polymerization. In the CP functionalization process, the CP surface may be modified by physical and chemical methods [20]. Biomolecules are used to drop in/on the CPs’ surface in the chemical modification method.

Bulk polymers often have a low surface area; thus, they often present a poor sensing performance such as long-time response and low sensitivity because the target biomarkers cannot or only slowly penetrate the CP matrix [21]. Therefore, the development of CP nanostructures has recently been introduced as a promising strategy for enhancing the efficiency of electrochemical biosensors. CP nanostructures in a wide range of dimensions, from 1D to 3D, including nanotubes, microspheres, nanofibers, nanobelts, nanorods, and nanowires, have been fabricated and employed for electrochemical biosensors. Due to their unique structures, these CP nanostructures provide a larger surface area and virus biomarkers can rapidly penetrate and interact with polymer bioreceptors, resulting in a fast response and high sensor signals [22,23]. CP nanostructures can be synthesized by two approaches, including template-based (hard-template and soft-template) and non-template techniques. However, the control of morphologies to obtain the nanometer size has still generated huge challenges, especially in large scale production [24]. CP nanowires, nanotubes, and microspheres are common nanostructures used for the development of highly sensitive electrochemical biosensors in virus detection [17]. The next strategy that has been applied to increase the stability of CP-electrochemical biosensors is the development of CP composites. Most pristine CPs show poor stability under different environmental conditions [25], but the integration of CPs with other conducting or insulating components can reduce their oxidation and degradation, as well as improve their charge transport properties [26]. Compared with pure CPs, their composites also exhibit superior advantages for the development of electrochemical biosensors, such as various morphologies, controllable electrochemical/chemical properties, and a larger edge plane/basal plane ratio [27]. CP composites are likely to be designed as excellent transducers in electrochemical biosensors for the detection of various virus biomarkers such as RNA, protein, antibodies, and whole virus. Carbon-based materials, metal nanoparticles, metal oxide nanoparticles, and other polymers have been used to make composites with CPs for the fabrication of electrochemical biosensors for virus detection.

As a promising strategy for the development of flexible and portable biosensors, CP hydrogels have recently been developed and employed for electrochemical biosensors. By taking advantage of both CPs and hydrogels, CP hydrogels exhibit outstanding features such as high electrical conductivity, good mechanical flexibility, high stretchability, biocompatibility, and ease of processing [28]. Moreover, CP hydrogels can enhance the sensing ability of bioreceptors in electrochemical biosensors due to several superior advantages: (i) providing a large interface area of CPs due to the 3D hydrogel structure; (ii) the soft property of the hydrogel matrix can easily immobilize biomolecular probes on the hydrogel surface; (iii) increasing the electron collection; (iv) decreasing the impedance [29,30,31]. Electrochemical biosensors that are constructed by CP hydrogels have been proven to show a higher sensing performance, high sensitivity, a low limit of detection (LOD), and a rapid response time compared to traditional CPs-based biosensors. For instance, Yang et al. reported a sensitive, rapid, and flexible biosensor based on polyaniline (PANI), a well-known CP, for diagnosing RNA biomarkers [32]. Due to their multiple pore structures, excellent electrochemical properties, and antifouling ability, the PANI hydrogel-based biosensors showed a high sensing performance towards a microRNA biomarker with a LOD of 0.34 fM. Therefore, CP hydro-gels can be considered as a potential approach for the development of flexible electrochemical biosensors in the detection of COVID-19 and other viruses.

In conclusion, using CPs to design and develop flexible and wearable biosensors has been one of the most important strategies in the early detection of COVID-19 and other viruses in the present and future. Moreover, integrating CP-based biosensors with the Internet of Things (IoT) can be used to increase the practical applications of CP-biosensors and provide an effective method of preventing virus infections, as well as providing greater ease of use for members of the community. This combination can allow the CP-based biosensor technology to significantly contribute to the prevention of the spread of COVID-19 and other viruses.

## Figures and Tables

**Figure 1:**
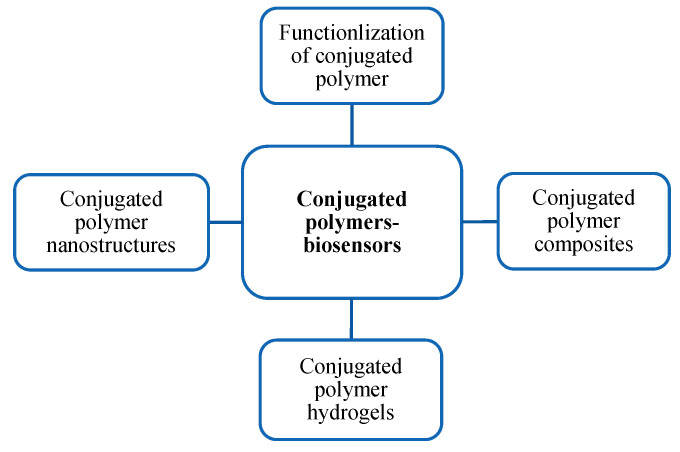
Development strategies of conjugated polymers-based biosensors for virus detection.

## Data Availability

The data presented in this study are available on request from the corresponding author.
